# Chemotherapy before or after preoperative chemoradiotherapy and surgery for locally advanced rectal cancer: 5-year results of the CAO/ARO/AIO-12 trial - a general pairwise comparison

**DOI:** 10.1016/j.esmoop.2025.104483

**Published:** 2025-03-07

**Authors:** M. Diefenhardt, R. Kosmala, M. Fleischmann, D. Martin, R.-D. Hofheinz, M. Ghadimi, C. Rödel, B. Polat, E. Fokas

**Affiliations:** 1 Goethe-University Frankfurt, University Hospital, Department of Radiotherapy, Frankfurt, Germany; 2 Frankfurt Cancer Institute, Frankfurt, Germany; 3 University Würzburg, University Hospital, Department of Radiation Oncology, Würzburg, Germany; 4 German Cancer Research Center (DKFZ), Heidelberg, Germany; 5 German Cancer Consortium (DKTK), Partner Site Frankfurt am Main, Frankfurt, Germany; 6 Department of Hematology and Oncology, University Medical Center Mannheim, Medical Faculty Mannheim, Heidelberg University, Mannheim, Germany; 7 University Göttingen, University Hospital, Department of General, Visceral and Pediatric Surgery, Göttingen, Germany; 8 University of Cologne, Faculty of Medicine and University Hospital Cologne, Department of Radiation Oncology, Cyberknife and Radiation Therapy, Cologne, Germany

**Keywords:** rectal cancer, general pairwise comparison, total neoadjuvant treatment, clinical trial, radiotherapy

## Abstract

**Background:**

Total neoadjuvant treatment (TNT) has been increasingly adopted for multimodal rectal cancer treatment. Here, we present the 5-year results of our CAO/ARO/AIO-12 randomized phase II trial that compared two TNT sequences.

**Patients and methods:**

Patients were initially randomized 1 : 1 to arm A (induction chemotherapy followed by chemoradiotherapy) or arm B (chemoradiotherapy followed by consolidation chemotherapy) followed by total mesorectal excision surgery. This report on the 5-year results involved a general pairwise comparison (GPC) of the following parameters, ranked as indicated: overall survival, incidence of locoregional recurrence, incidence of distant metastasis, rate of pathological/clinical complete remission, long-term quality of life (at least 24 months after randomization) based on global health assessed by the European Organisation For Research And Treatment Of Cancer Quality of Life Questionnaire Core 30 questionnaire, and incidence of toxicity, ranked by grade, during follow-up.

**Results:**

A total of 306 patients were eligible for this analysis. After a median follow-up of 60 months (interquartile range 58-62 months), we found that long-term oncological outcome was comparable in both arms [e.g. 5-year overall survival 85.8% (95% confidence interval 80.2% to 91.8%) in arm A and 84.2% (95% confidence interval 78.2% to 90.5%) in arm B], regardless of whether patients received induction chemotherapy and chemoradiotherapy or chemoradiotherapy and consolidation chemotherapy. The GPC showed no clinically meaningful overall treatment benefit (−1.38%) or win ratio difference (0.97) between the two treatment sequences. The incidence of pathological or sustained clinical complete remission remained higher in patients treated with consolidation chemotherapy after adjusting for long-term outcome between both arms (11% versus 6.5%).

**Conclusions:**

Our 5-year GPC confirmed the 3-year findings that chemoradiotherapy followed by consolidation chemotherapy resulted in higher rates of pathological complete remission without compromising oncological outcome, toxicity, or quality of life. The TNT sequence chemoradiotherapy/chemotherapy may be preferred for organ preservation strategies.

## Highlights

• Five-year results of the clinical phase II CAO/ARO/AIO-12 trial.
• The general pairwise comparison: an innovative method to analyze and report trial results in rectal cancer.
• Higher pCR after TNT with consolidation chemotherapy did not improve long-term survival, toxicity or QoL.
• The acquisition of long-term data on quality of life, in particular on sexual functioning, must be improved.
• Consolidation chemotherapy after chemoradiation is an attractive option for organ-preserving strategies in rectal cancer.

## Introduction

Total neoadjuvant treatment (TNT) can be considered a new standard of care in patients with locally advanced rectal cancer with high-risk features after the PRODIGE-23 trial and the RAPIDO trial reported higher incidence of pathological complete remission (pCR) after TNT with improved disease-free survival (DFS) and even overall survival (OS) in the 7-year report of the PRODIGE-23 trial^1^, ^2^, ^3^, ^4^ without significantly compromising long-term health-related quality of life (QoL).^5^

The CAO/ARO/AIO-12 trial and the OPRA trial are the only randomized trials that compared two TNT sequences (induction versus consolidation chemotherapy before or after chemoradiotherapy).^6^^,^^7^ The primary endpoints results of both trials have already been reported.^6^, ^7^, ^8^, ^9^ The CAO/ARO/AIO-12 trial demonstrated a higher rate of pCR in favor of consolidation chemotherapy, with comparable DFS between the two arms after a median follow-up of 43 months.^7^^,^^8^ The OPRA trial found no significant differences in DFS after a 5-year median follow-up. Total mesorectal excision (TME)-free survival, however, was 39% [95% confidence interval (CI) 32% to 48%] after induction chemotherapy followed by chemoradiation and 54% (95% CI 46% to 62%) after chemoradiation followed by consolidation chemotherapy (*P* = 0.012).^9^^,^^10^

The general pairwise comparison (GPC) of prioritized outcomes is a statistical approach to analyze multiple outcomes (survival, toxicity, QoL), regardless of their type (time, binary, continuous) in a single formal analysis, resulting in a general measure of the differences between two treatment arms.^11^ The major advantage of this analysis is that the order of priorities can be adjusted for individual patients, allowing clinicians to interpret trial data in a more personalized, patient-centered way and to directly analyze the potential impact of toxicity or deteriorating of QoL on an potential long-term survival benefit.^12^

Using GPC analysis, we report here 5-year follow-up data from the CAO/ARO/AIO-12 trial with updated safety and patient-reported outcomes (PROs) for QoL over the entire long follow-up period.

## Patient and methods

### Study design and participants

The CAO/ARO/AIO-12 trial, registered with ClinicialTrials.gov (NCT02363374), was an open-label, multicenter, randomized phase II trial in patients with locally advanced rectal cancer. Details of the design of the CAO/ARO/AIO-12 trial have been previously published.^7^ Patients were eligible if they were at least 18 years old and had histologically confirmed rectal adenocarcinoma up to 12 cm above the anal verge based on rigid rectoscopy. Magnetic resonance imaging was mandatory, and patients were eligible if they met one of the following criteria: cT3 tumor <6 cm from the anal verge, cT3 tumor in the middle third of the rectum (≥6 to 12 cm) with extramural tumor spread into the mesorectal fat of >5 mm (>cT3b), cT4 tumor, or lymph node involvement. Other inclusion criteria were Eastern Cooperative Oncology Group performance status 0 or 1 and adequate organ function. Abdomen/chest computed tomography was carried out to exclude distant metastases (DM).

CAO/ARO/AIO-12 was conducted in accordance with the Declaration of Helsinki and Good Clinical Practice guidelines. The study protocol and amendments were approved by the institutional review boards or independent ethics committees at each study site, and all patients gave written informed consent before enrolment.

### Procedures

Patients were randomly assigned to either arm A for induction chemotherapy before chemoradiotherapy or arm B for consolidation chemotherapy after chemoradiotherapy. Preoperative radiotherapy consisted of 50.4 Gy in 28 fractions using intensity-modulated radiotherapy to the primary tumor and the mesorectal, presacral, and internal iliac lymph nodes. Concurrent chemotherapy was administered with a continuous infusion of fluorouracil (250 mg/m^2^) on days 1 to 14 and 22 to 35 and a 2-h infusion of oxaliplatin (50 mg/m^2^) on days 1, 8, 22, and 29 of radiotherapy. Induction/consolidation chemotherapy consisted of oxaliplatin (100 mg/m^2^) administered as a 2-h infusion, followed by a 2-h infusion of leucovorin (400 mg/m^2^), followed by a continuous 46-h infusion of fluorouracil (2400 mg/m^2^), repeated on day 15 for a total of three cycles. TME surgery was scheduled for day 123 after the start of TNT. Surgery was indicated regardless of tumor response; no watch and wait strategy was intended.

### Outcomes

The primary endpoint, pCR,^7^ and the 3-year results for secondary endpoints including survival and toxicity after a median follow-up of 43 months have been published before.^8^ Here we report 5-year results regarding survival, toxicity, and QoL assessed by the European Organisation For Research And Treatment Of Cancer (EORTC) Quality of Life Questionnaire Core 30 (QLQ-C30) and the colorectal cancer-specific module CR29 (http://www.eortc.be/home/qol) in a comprehensive GPC.

### Statistical analyses

**Figure 1:**
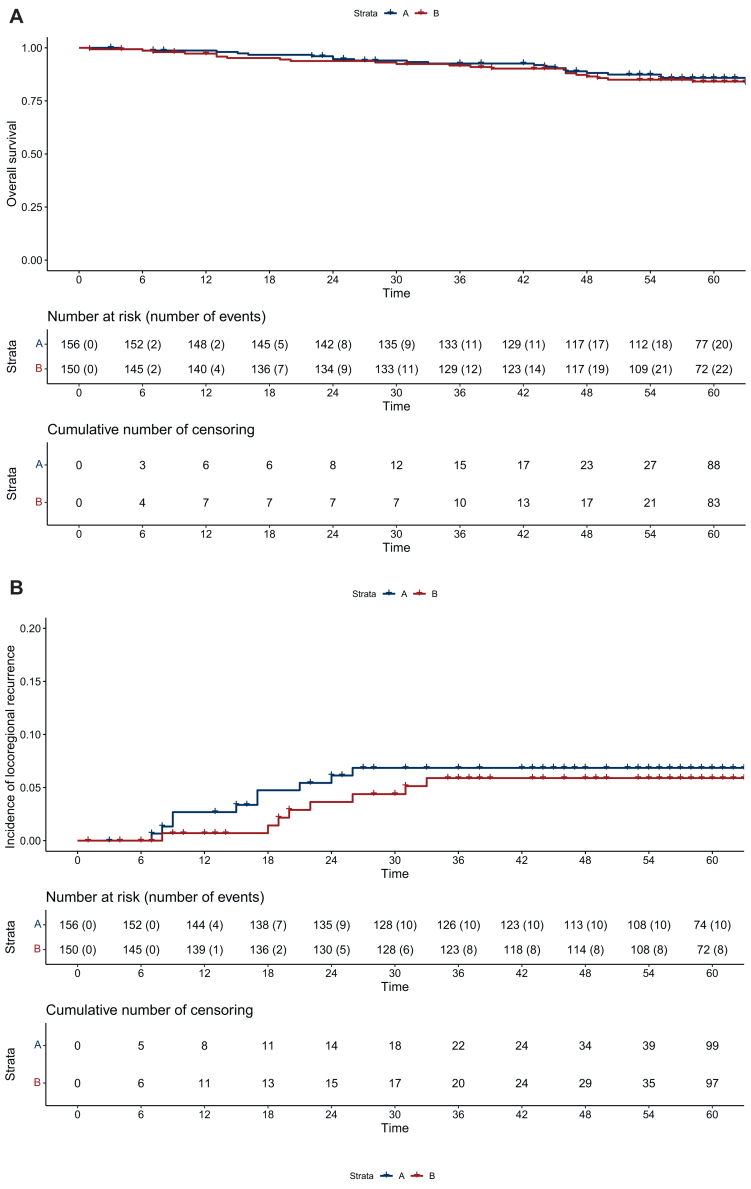

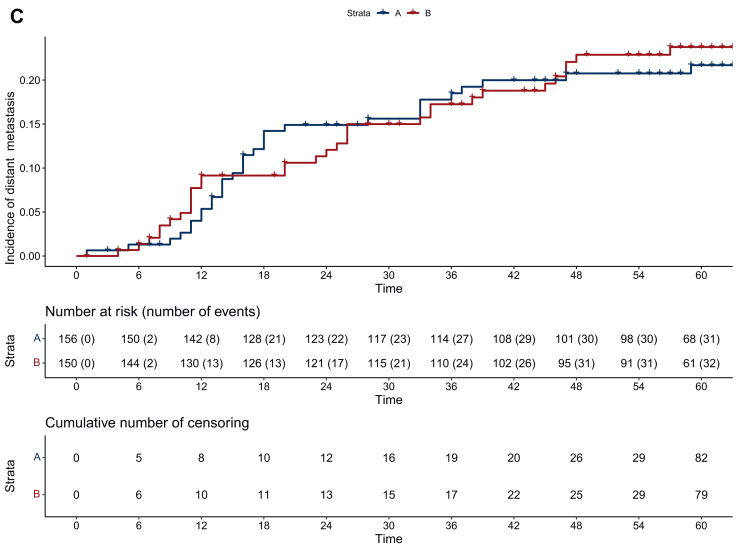
**Long-term oncologic outcomes.** (A) Overall survival in arm A and arm B, (B) cumulative incidence of locoregional recurrence in arm A and arm B and (C) incidence of distant metastasis in arm A and arm B. CI, confidence interval; NTB, net treatment benefit; pCR, pathological complete remission.

A GPC was carried out to analyze differences between the two treatment arms according to Buyse.^11^^,^^13^ GPC can compare multiple outcomes, however the priority of the outcomes of interest, e.g. if OS is more important than pCR, must be decided before the analyses. Patients from both TNT arms are paired by random, and their outcomes for the endpoint of interest are compared. If a patient has a better outcome in terms of the endpoint of interest, that patient is classified as a winner in its treatment arm. A pair that shows no difference in the endpoint of interest (‘neutral’), or if it cannot be determined due to missing values (‘uninformative’), is moved to the next lower prioritized endpoint for comparison, with both possibilities referred to as ‘tie’ in Figure 1. This process is repeated for all ranked endpoints. Finally, all comparisons are statistically combined into a summary measure that allows the calculation of a net treatment benefit, which describes the probability that a patient will have a better outcome in one treatment arm compared with the other arm. The endpoints in this analysis were selected and ranked by the authors after internal discussion. OS, locoregional recurrence (LR) and DM were analyzed as time-to-event, complete remission [pathological complete remission/clinical complete remission (cCR)] as a binary variable and QoL and toxicity as ordinal parameters. For OS and the incidence of LR and DM, a difference of 1 month was considered clinically relevant. This implies that in our analysis, pairs of patients with censoring or the same outcome, or if both patients in a pair died or had LR or DM with <1 month difference, were considered neutral and no ‘winner’ or ‘loser’ was set.^11^^,^^14^ Long-term outcomes, toxicity, and QoL outcomes are also reported for the full cohort. QoL analyses using the EORTC QLQ-30 and CR29 questionnaires were not restricted to disease-free patients, but following the principles of the stepwise GPC approach, most patients with locoregional recurrence and/or DM were already defined as ‘winner’ or ‘loser’ in the higher ranked endpoints. After linear transformation, the changes in QoL reported in Table 1, Table 2 were calculated without restriction to potentially meaningful thresholds of clinical significance.^15^ Changes in functional or symptom health-related QoL scores were analyzed only in patients with complete information for specific symptom or functional scores at baseline and after at least 24 months of follow-up, respectively, for the latest available follow-up visit, and independently in male and female patients. A *P* value <0.05 was considered statistically significant. Due to the exploratory nature of this analysis, no adjustment for multiple testing was made. All analyses were carried out in R statistic software.^13^

**Table 1:** Toxicities reported during the follow-up period, classified according to the CTCAE version 4 by treatment arm

Toxicity—CTCAE Version 4	Treatment arm A								Treatment arm B								Relative differences between arm B and arm A (%)	
					Total number/percentage of all patients *n* = 156								Total number/percentage of all patients *n* = 150					
	1	2	3	4	All grades		High grades		1	2	3	4	All grades		High grades		All grades	High grades
Oxaliplatin-induced neurotoxicity according to Wassermann score	32	14	29	2	77	49	31	20	32	16	27	1	76	51	28	19	+2	−1
Sensorium	49	15	5	0	69	44	5	3	43	16	3	0	62	41	3	2	−3	−1
Diarrhea	35	21	5	0	61	39	5	3	42	21	7	0	70	47	7	5	+8	+2
Fecal incontinence	18	15	5	0	38	24	5	3	20	14	5	0	39	26	5	3	+2	+0
Erectile dysfunction	9	11	3	2	25	16	5	3	9	9	10	3	31	21	13	9	**+5**	+6
Bladder emptying disorder	18	3	1	0	22	14	1	1	10	3	2	0	15	10	2	1	−4	+0
Proctitis	11	2	1	0	14	9	1	1	17	4	2	0	23	15	2	1	+6	+0
Cystitis (non-infectious)	11	1	1	0	13	8	1	1	15	2	0	0	17	11	0	0	+3	−1
Skin (radiation-induced dermatitis)	10	3	0	0	13	8	0	0	4	2	0	0	6	4	0	0	−4	+0
Anastomotic stenosis	3	4	2	0	9	6	2	1	5	4	3	0	12	8	3	2	+2	+1
Small intestinal fistula	0	0	2	0	2	1	2	1	1	0	0	0	1	1	0	0	+0	−1
Fistula to the urinary bladder	0	0	1	0	1	1	1	1	1	0	0	0	1	1	0	0	+0	−1
Rectovaginal fistula	0	0	1	0	1	1	1	1	2	0	2	0	4	3	2	1	+2	+0
Vaginal dryness	1	1	0	0	2	1	0	0	1	1	2	0	4	3	2	1	+2	+1
Highest toxicity per patient	30	38	48	4	120	77	52	33	33	33	45	4	115	77	49	33	+0	+0

CTCAE, Common Terminology Criteria for Adverse Events.

**Table 2:** Change in quality of life between baseline and the last available follow-up visit, but at least 24 months after randomization, according to the EORTC QLQ-C30 questionnaire by treatment arm

EORTC QLQ-C30		Treatment arm A—CT CRT				Treatment arm B—CRT CT				*P* value	Commentary
		Missing	Negative, *n* (%)	Neutral, *n* (%)	Positive, *n* (%)	Missing, *n* (%)	Negative, *n* (%)	Neutral, *n* (%)	Positive, *n* (%)		
Global health	Male	43	20 (32)	13 (21)	30 (48)	41	18 (31)	17 (29)	24 (41)	0.88	In 54 of 122 (44%) men global health improved.
	Female	23	10 (37)	6 (22)	11 (41)	21	12 (41)	5 (17)	12 (41)	0.99	In 23 of 56 (41%) women global health improved.
Physical functioning	Male	44	37 (60)	16 (26)	9 (15)	40	34 (57)	21 (35)	5 (8)	0.51	In 71 of 122 (58%) men physical functioning worsened.
	Female	25	17 (68)	7 (28)	1 (4)	22	17 (61)	4 (14)	7 (25)	0.97	In 34 of 53 (64%) women physical functioning worsened.
Role functioning	Male	42	31 (48)	20 (31)	13 (20)	40	27 (45)	22 (37)	11 (18)	0.58	In 58 of 124 (47%) men role functioning worsened.
	Female	23	17 (63)	5 (19)	5 (19)	21	14 (48)	8 (28)	7 (24)	0.61	In 31 of 56 (55%) women role functioning worsened.
Emotional functioning	Male	45	19 (31)	6 (10)	36 (59)	40	17 (28)	17 (28)	26 (43)	0.77	In 62 of 121 (51%) men emotional functioning improved.
	Female	22	9 (32)	4 (14)	15 (54)	22	6 (21)	5 (18)	17 (61)	0.88	In 32 of 56 (57%) women emotional functioning improved.
Cognitive functioning	Male	45	24 (39)	29 (48)	8 (13)	40	23 (38)	29 (48)	8 (13)	0.73	In 58 of 121 (48%) men cognitive functioning did not change.
	Female	25	8 (32)	12 (48)	5 (10)	23	12 (44)	11 (41)	4 (15)	**0.04**	In 23 of 52 (44%) women cognitive functioning did not change.
Social functioning	Male	44	28 (45)	22 (35)	12 (19)	43	23 (40)	21 (37)	13 (23)	0.49	In 51 of 119 (43%) men social functioning worsened.
	Female	23	14 (52)	7 (26)	6 (22)	23	11 (41)	7 (26)	9 (33)	0.78	In 25 of 54 (46%) women social functioning worsened.
Fatigue	Male	43	29 (46)	13 (21)	21 (33)	41	22 (37)	20 (34)	17 (29)	0.30	In 51 of 122 (42%) men fatigue worsened.
	Female	24	13 (50)	8 (31)	5 (19)	23	9 (33)	9 (33)	9 (33)	0.73	In 21 of 53 (40%) women fatigue worsened.
Nausea and vomiting	Male	43	8 (13)	54 (86)	1 (2)	39	9 (15)	50 (82)	2 (3)	0.51	In 104 of 124 (84%) men nausea did not change.
	Female	22	3 (11)	23 (82)	2 (7)	21	5 (17)	20 (69)	4 (14)	0.79	In 43 of 57 (75%) women nausea did not change.
Pain	Male	43	24 (38)	28 (44)	11 (17)	41	16 (28)	28 (48)	14 (24)	0.34	In 56 of 121 (46%) men pain did not change.
	Female	22	15 (54)	8 (29)	5 (18)	22	6 (21)	15 (54)	7 (25)	0.05	In 23 of 56 (41%) women pain did not change.
Dyspnea	Male	44	21 (34)	36 (58)	5 (8)	40	19 (32)	36 (60)	5 (8)	0.63	In 72 of 122 (59%) men dyspnea did not change.
	Female	22	12 (43)	16 (57)	0 (0)	21	10 (34)	19 (66)	0 (0)	0.78	In 35 of 57 (61%) women dyspnea did not change.
Insomnia	Male	42	25 (39)	27 (42)	12 (19)	60	15 (38)	23 (58)	2 (5)	0.07	In 50 of 104 (48%) men insomnia did not change.
	Female	22	6 (21)	17 (61)	5 (18)	21	7 (24)	16 (55)	6 (21)	0.85	In 33 of 57 (58%) women insomnia did not change.
Appetite loss	Male	42	10 (16)	47 (73)	7 (11)	39	6 (10)	42 (69)	13 (21)	0.09	In 89 of 125 (71%) men appetite did not change.
	Female	23	3 (11)	21 (78)	3 (11)	22	4 (14)	18 (64)	6 (21)	0.99	In 39 of 55 (71%) women appetite did not change.
Constipation	Male	44	11 (18)	40 (65)	11 (18)	40	16 (27)	33 (55)	11 (18)	0.48	In 73 of 122 (60%) men appetite did not change.
	Female	22	4 (14)	21 (75)	3 (11)	21	4 (14)	21 (72)	4 (14)	0.45	In 42 of 57 (74%) women appetite did not change.
Diarrhea	Male	43	15 (24)	23 (37)	25 (40)	41	19 (32)	14 (24)	26 (44)	0.26	In 51 of 122 (42%) men diarrhea health improved.
	Female	22	8 (29)	9 (32)	11 (39)	21	13 (45)	12 (41)	4 (14)	0.06	In 22 of 57 (39%) women diarrhea did not change.
Financial difficulties	Male	43	15 (24)	44 (70)	4 (6)	42	16 (28)	35 (60)	7 (12)	0.63	In 79 of 121 (65%) men financial difficulties did not change.
	Female	23	9 (33)	16 (59)	2 (7)	23	6 (22)	16 (59)	5 (19)	**0.04**	In 32 of 54 (60%) women financial difficulties did not change.

The *P* value refers to the results of the *t*-test for QoL in arm A compared to arm B in male and female patients, respectively. *P* < 0.05 are indicated in bold.

CRT, chemoradiotherapy; CT, chemotherapy; EORTC QLQ-C30, European Organisation For Research And Treatment Of Cancer Quality of Life Questionnaire Core 30; QoL, quality of life.

## Results

### Baseline characteristics and patient disposition

Between June 2015 and January 2018, 311 patients were enrolled, of whom 306 were eligible for this analysis: 156 in arm A and 150 in arm B, as previously described. Baseline patient demographics and clinical characteristics have been previously reported.^7^

### Efficacy

**Figure 2:**
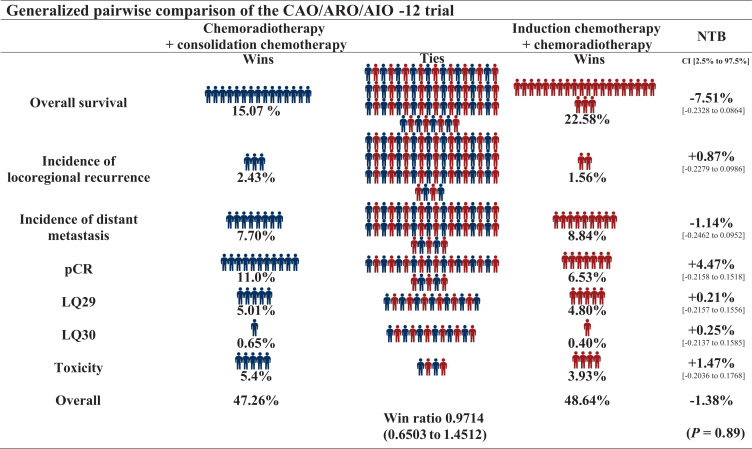
**General pairwise comparison (GPC) net treatment benefit (NTB) and win ratio refers to arm B (consolidation chemotherapy)**.

After induction chemotherapy followed by chemoradiotherapy, the rate of complete remission (pCR and sustained cCR in the case the patients rejected surgery) was 21% (32 of 156 patients) in arm A compared with 27% (41 of 150 patients) after chemoradiotherapy followed by consolidation chemotherapy (GPC delta 0.0682, 95% CI −0.0275 to 0.1627, *P* = 0.16). After a median follow-up of 60 months (interquartile range 58-62 months), 5-year OS was 85.8% (95% CI 80.2% to 91.8%) in arm A and 84.2% (95% CI 78.2% to 90.5%) in arm B (GPC delta −0.071, 95% CI −0.2328 to 0.0864, *P* = 0.36, Figure 2A), 5-year LR incidence was 6.9% (95% CI 2.67% to 10.87%) in arm A and 5.9% (95% CI 1.85% to 9.78%) in arm B (GPC delta 0.0116, 95% CI −0.0453 to 0.0685, *P* = 0.69, Figure 2B), and the 5-year incidence of DM was 21.7% (95% CI 14.59% to 28.21%) in arm A and 23.76% (95% CI 16.17% to 30.66%) in arm B (GPC delta −0.0162, 95% CI −0.1152 to 0.0831, *P* = 0.75, Figure 2C).

### Safety

During the 5-year follow-up period, toxicity of any grade according to the Common Terminology Criteria for Adverse Events (CTCAE) version 4.0 classification was reported in 120 patients (77%) in arm A and in 115 patients (77%) in arm B. Higher-grade toxicity (CTCAE ≥grade 3) was reported in 52 patients (33%) in arm A and 49 patients (33%) in arm B. Patients in arm B were more likely to report diarrhea of any grade (39% in arm A versus 47% in arm B), erectile dysfunction of any grade and higher grade (16%/3% in arm A versus 21%/9% in arm B), and proctitis of any grade (9% in arm A versus 15% in arm B) on at least one follow-up visit. Overall, oxaliplatin-associated peripheral sensory neuropathy/paresthesia was reported in 49% of patients in arm A and 51% of patients in arm B (Table 1). The GPC showed no significant differences in the overall toxicity profile between the two treatment arms (delta 0.0176, 95% CI −0.1074 to 0.142, *P* = 0.78).

### QoL

Overall, information on long-term development of PROs was available for ∼60% of patients. Global health status improved in both treatment arms, with 43% of patients reporting an improvement. A GPC between arm A and arm B for question 29, *How would you rate your overall health during the past week?* (delta 0.024, 95% CI −0.0404 to 0.0882, *P* = 0.47) and question 30, *How would you rate your overall quality of life during the past week?* (delta 0.0319, 95% CI −0.0322 to 0.0957, *P* = 0.33) of the EORTC C30 questionnaire showed no significant differences between the two sequences. In addition to global health, a relative majority of patients tended to report improvements in emotional functioning (53% of patients), diarrhea (37%), blood and mucus in stools (76%), and anxiety (66%). In contrast, deterioration in health-related QoL scores was mainly reported for physical functioning (in 60% of patients), role functioning (49%), social functioning (44%), fatigue (42%), urinary frequency (40%), and body image (52%). Analyses of the effect of treatment on sexual function are limited by the small number of responses available. In arm A 20 men (43%) and in arm B 29 men (63%) reported a worsening of impotence, whereas in arm A 1 (14%) woman and in arm B 6 (46%) women reported a worsening of dyspareunia. Two-sided *t*-tests not adjusted for multiple testing indicated a potential difference in the change of health-related QoL scores in women assessing hair loss (worsening in 44% arm A versus 12% arm B, *P* = 0.04), cognitive function (worsening in 32% arm A versus 44% arm B, *P* = 0.04) and financial difficulties (worsening in 33% arm A versus 22% arm B, *P* = 0.04) (Table 2, Table 3).

**Table 3:** Change in quality of life between baseline and the last available follow-up visit, but at least 24 months after randomization, according to the EORTC QLQ-CR29 questionnaire by treatment arm

EORTC QLQ-CR29		Treatment Arm A—CT CRT				Treatment Arm B—CRT CT				*P* value	Commentary
		Missing	Negative, *n* (%)	Neutral, *n* (%)	Positive, *n* (%)	Missing	Negative, *n* (%)	Neutral, *n* (%)	Positive, *n* (%)		
Urinary frequency	Male	48	19 (33)	19 (33)	20 (34)	46	26 (48)	13 (24)	15 (28)	0.18	In 45 of 112 (40%) men urinary frequency worsened.
	Female	26	7 (29)	12 (50)	5 (19)	23	13 (48)	4 (15)	10 (37)	0.97	In 20 of 51 (39%) women urinary frequency worsened.
Blood and mucus in stool	Male	51	2 (4)	12 (22)	41 (74)	48	2 (4)	8 (15)	42 (81)	0.51	In 83 of 107 (78%) men blood and mucus in stool improved.
	Female	28	1 (5)	6 (27)	15 (68)	24	1 (4)	5 (21)	18 (75)	0.41	In 33 of 46 (72%) women blood and mucus in stool improved.
Body image	Male	57	27 (55)	17 (35)	5 (10)	48	25 (48)	18 (35)	9 (17)	0.91	In 52 of 101 (51%) men body image worsened.
	Female	27	13 (57)	6 (26)	4 (17)	21	11 (50)	6 (27)	5 (24)	0.88	In 24 of 45 (53%) women body image worsened.
Urinary incontinence	Male	50	11 (20)	42 (75)	3 (5)	51	10 (20)	35 (71)	4 (8)	0.90	In 77 of 105 (73%) men urinary incontinence did not change.
	Female	26	4 (17)	18 (75)	2 (8)	24	10 (38)	13 (50)	3 (12)	0.28	In 31 of 50 (62%) women urinary incontinence did not change.
Dysuria	Male	47	8 (14)	47 (80)	4 (7)	57	5 (9)	47 (82)	5 (9)	0.43	In 94 of 116 (81%) men dysuria did not change.
	Female	26	1 (4)	25 (96)	0 (0)	21	2 (7)	25 (86)	2 (7)	0.25	In 50 of 55 (91%) women dysuria did not change.
Abdominal pain	Male	49	15 (26)	38 (67)	4 (7)	43	9 (16)	41 (72)	7 (12)	0.17	In 79 of 114 (69%) men abdominal pain did not change.
	Female	25	6 (24)	15 (60)	4 (16)	23	3 (11)	17 (63)	7 (26)	0.04	In 32 of 52 (62%) women abdominal pain did not change.
Buttock pain	Male	48	14 (24)	25 (43)	19 (33)	55	13 (24)	22 (40)	20 (36)	0.92	In 47 of 113 (42%) men buttock pain did not change.
	Female	24	8 (31)	12 (46)	6 (23)	27	7 (26)	13 (48)	7 (26)	0.79	In 25 of 53 (47%) women buttock pain did not change.
Bloated feeling	Male	47	11 (19)	32 (54)	16 (27)	43	16 (28)	27 (47)	14 (25)	0.20	In 59 of 116 (51%) men bloated did not change.
	Female	26	9 (38)	10 (42)	5 (21)	24	7 (27)	12 (46)	7 (27)	0.18	In 22 of 50 (44%) women bloated did not change.
Dry mouth	Male	48	15 (26)	33 (57)	10 (17)	58	19 (33)	28 (48)	11 (19)	0.47	In 61 of 116 (53%) men dry mouth did not change.
	Female	25	6 (24)	16 (64)	3 (12)	25	5 (20)	12 (48)	8 (32)	0.18	In 28 of 50 (56%) women dry mouth did not change.
Hair loss	Male	49	12 (21)	45 (79)	0 (0)	54	10 (19)	43 (80)	1 (2)	0.97	In 88 of 111 (79%) men hair loss did not change.
	Female	25	11 (44)	12 (48)	2 (8)	25	3 (12)	20 (80)	2 (8)	**0.04**	In 32 of 50 (64%) women hair loss did not change.
Trouble with taste	Male	47	9 (15)	47 (80)	3 (5)	56	9 (16)	45 (80)	2 (4)	0.60	In 92 of 115 (80%) men hair loss did not change.
	Female	25	4 (16)	20 (80)	1 (4)	25	4 (16)	19 (76)	2 (8)	0.61	In 39 of 50 (78%) women hair loss did not change.
Anxiety	Male	48	5 (9)	15 (26)	38 (66)	59	7 (12)	15 (25)	37 (63)	0.73	In 75 of 117 (64%) men anxiety improved.
	Female	25	4 (16)	5 (20)	16 (64)	27	0 (0)	5 (19)	22 (81)	0.33	In 38 of 52 (73%) women anxiety improved.
Flatulence	Male	53	18 (35)	27 (52)	8 (15)	45	23 (51)	15 (33)	7 (16)	0.45	In 42 of 98 (80%) men flatulence did not change.
	Female	30	8 (40)	7 (35)	5 (25)	21	9 (43)	10 (48)	2 (10)	0.36	In 17 of 41 (41%) women flatulence did not change.
Fecal incontinence	Male	52	18 (33)	22 (41)	14 (26)	44	19 (43)	19 (43)	6 (14)	0.19	In 41 of 98 (42%) men fecal incontinence did not change.
	Female	30	6 (30)	11 (55)	3 (15)	21	8 (38)	11 (52)	2 (10)	0.32	In 22 of 41 (54%) women hair loss did not change.
Score skin	Male	52	20 (37)	23 (43)	11 (21)	43	17 (40)	19 (44)	7 (16)	0.83	In 42 of 97 (43%) men skin did not change.
	Female	30	5 (25)	10 (50)	5 (25)	25	10 (40)	12 (48)	3 (12)	0.41	In 22 of 45 (49%) women skin did not change.
Embarrassment	Male	54	17 (33)	25 (48)	10 (19)	38	20 (53)	14 (37)	4 (11)	0.12	In 39 of 97 (40%) men embarrassment did not change.
	Female	32	5 (28)	10 (56)	3 (17)	24	7 (29)	12 (50)	5 (21)	0.70	In 22 of 42 (53%) women embarrassment did not change.
Sexual interest	Male	55	15 (29)	26 (51)	10 (20)	54	16 (27)	22 (37)	16 (27)	0.38	In 48 of 105 (46%) men sexual interest did not change.
	Female	33	6 (35)	7 (41)	4 (24)	17	4 (24)	10 (59)	3 (18)	0.88	In 17 of 34 (50%) women sexual interest did not change.
Impotence	Male	60	20 (43)	19 (41)	7 (15)	46	29 (63)	13 (28)	4 (9)	0.13	In 49 of 92 (53%) men impotence worsened.
Dyspareunia	Female	43	1 (14)	4 (57)	2 (29)	13	6 (46)	6 (46)	1 (8)	0.10	In 10 of 20 (50%) women dyspareunia did not change.

The *P* value refers to the results of the *t*-test for QoL in arm A compared to arm B in male and female patients, respectively. *P* < 0.05 are indicated in bold.

CRT, chemoradiotherapy; CT, chemotherapy; EORTC QLQ-C30, European Organisation For Research And Treatment Of Cancer Quality of Life Questionnaire Core 30.

### General pairwise comparison

At the authors’ discretion, the following outcome parameters were analyzed in a GPC, ranked as indicated: OS, incidence of LR, incidence of DM, rate of remission (pCR/cCR), long-term QoL (at least 24 months after randomization) based on EORTC QLQ-C30 questions 29 and 30, and incidence of toxicity classified on CTCAE version 4.0 classification during follow-up.

The GPC approach showed no statistically significant differences between the two treatment arms. The probability of having a better outcome with CRT and consolidation chemotherapy was 47.26% compared with a 48.64% probability of having a better outcome with induction chemotherapy followed by CRT. The net treatment benefit was −1.38%, which correlated with a win ratio (*the win ratio is the total number of winners divided by the total numbers of losers*,^16^ *equal to 1 if there is no treatment effect*^17^) of 0.97 (95% CI 0.6503-1.4512, *P* = 0.89) (Figure 2). After eliminating patient pairs without the same long-term oncological outcome, the probability of complete remission remained higher at 11% after consolidation versus 6.5% after induction chemotherapy. The difference in global health and toxicity indicated no clinically meaningful difference in patients with comparable short-term (response to TNT) and long-term outcome.

## Discussion

The 5-year analysis of the CAO/ARO/AIO-12 trial showed no clinically meaningful long-term differences between the two TNT sequences. After a median follow-up of 60 months, the initial better tumor response to consolidation chemotherapy did not translate into a better long-term oncological outcome, superior toxicity profile or better health-related QoL. While symptoms such as diarrhea, blood and mucus in the stool, and emotional functioning improved, other domains/symptoms such as fatigue, impotence in male, physical and role functioning worsened in both arms. The improvement in tumor-related symptoms after treatment is consistent with a report from the Prodige-23 trial where they reported improved symptoms after induction chemotherapy. Our analysis did not support their conclusion, however, apparently based on different TNT regimes, that the reduction in functional scores can be considered only transient.^18^ With regard to possible differences between the two treatment sequences, the limitation of *P* values in multiple testing due to the increasing likelihood of a type 1 error should be considered, and therefore any differences should be interpreted not only in terms of statistical significance, but also in terms of the actual effect size.^19^

Although we did not find clinically meaningful differences between the two treatment regimens, the overall heterogeneity in long-term QoL has to be considered in advising our patients.

Sexual function is an important long-term endpoint for patients but is often underestimated by clinicians.^20^ PROs from the PROSPECT trial showed better sexual function in men and women after neoadjuvant FOLFOX versus 5-FU chemoradiotherapy and surgery at 24 months.^21^ Unfortunately, only 205 patients were available for this long-term analysis. This represents only 22% of all patients who contributed PRO-CTCAE data and only 18% of patients who started treatment. In the RAPIDO trial, information on sexual interest at 36 months was available for 439 of 912 (48%) initial eligible patients, respectively, for 439 of 701 disease-free patients (63%) but as analyses of sexual function was restricted to patients with sexual activity these analyses were based only on 215 (24% of initial eligible patients, 31% of disease-free patients).^5^ In the PRODIGE-23 study, information on sexual interest or sexual function at 24 months was available for 136 (30%) and 128 (28%) of the 461 randomized patients, respectively, for 136 (40%) and 128 (37%) of 343 disease-free patients.^18^ In our study, sexual function data to compare baseline and at least 24 months follow-up were available in 37% of patients, but only in 20% of female patients. Therefore, in all these recent rectal cancer trials, the number of patients with long-term information on sexual activity and functioning is limited, so any conclusions based on these data should be treated with caution.

OS is still considered a gold standard in cancer clinical trials but whether it still represents the most appropriate endpoint for all clinical situations and trials has been questioned for years.^22^^,^^23^ In rectal cancer, DFS, TME-free survival or rate of organ preservation have been proposed as realistic primary endpoints to show clinically meaningful differences between two treatment strategies and still provide a robust association on long-term survival.^24^, ^25^, ^26^ These endpoints, however, may not always represent the most important endpoints from a patient perspective. In addition, composite endpoint analyses are often limited to a patient’s first event and consider all contributing events as equally relevant to a patient.^17^ In this context, GPC could provide important additional information for the interpretation of clinical trials. On the one hand, GPC allows us to analyze several endpoints with different priorities in one analysis. The ranking of the endpoint can be adjusted to individual patient priorities.^12^ Another main advantage of GPC is that it can show that achieving a survival benefit may come at a cost, and therefore the clinically meaningful benefit for patients may be limited, e.g. in case of an OS benefit that is, however, associated with a persistent deterioration in QoL. Also, GPC could identify a meaningful difference between two treatments even if the single endpoint did not reach a statistically significant level, e.g. if a treatment prolongs OS and provides a better toxicity profile than the alternative treatment.^11^ As no subgroup of patients was identified in the CAO/ARO/AIO-12 trial, and in the pooled analysis of the CAO/ARIO/AIO-12 trial with the OPRA trial, who were more likely to benefit from one or the other treatment sequence, GPC may be useful as it provides the flexibility to prioritise outcomes and thresholds for individual benefit-risk assessment.^7^^,^^14^^,^^27^, ^28^, ^29^

Our study has several limitations. First, this was a *post hoc* analysis and no adjustment for multiple testing was implemented. Second, endpoints were ranked only at the discretion of the authors. Rectal cancer patients, although they tended to rank longer survival as the most important outcome in the direct ranking, do not seem to accept longer survival at any cost. Van der Valk et al.^30^ reported in their questionnaire of 94 patients that avoidance of surgery with permanent stoma was ranked by patients as a higher priority than survival in a conjoint analysis. They also showed a valid imbalance in the prioritization of outcomes between clinicians and patients. Determining a meaningful survival benefit threshold for which most patients would accept experiencing higher-grade treatment-related toxicity or deterioration of QoL, however, can be very challenging.^14^ After a median follow-up of 5.1 years, the OPRA group reported a TME-free survival of 54% (95% CI 46% to 62%) with chemoradiotherapy followed by chemotherapy, but no detailed report on the potential QoL benefits for these patients has been published to date.^9^ In the CAO/ARO/AIO-12 trial, surgery after TNT was mandatory per protocol, so we cannot provide data on TME-free survival.

The 5-year analyses as a GPC of the CAO/ARO/AIO-12 study showed no significant differences in survival or clinically meaningful differences in long-term changes in health-related QoL between two TNT sequences. New strategies to improve the collection of long-term information on patient QoL are needed. Prioritized analysis of multiple endpoints in a GPC approach should be considered as a possible innovative primary endpoint in the era of organ preservation after TNT in locally advanced rectal cancer.
